# Altered cardiac mitochondrial dynamics and biogenesis in rat after short-term cocaine administration

**DOI:** 10.1038/s41598-021-03631-y

**Published:** 2021-12-16

**Authors:** Shuheng Wen, Kana Unuma, Takeshi Funakoshi, Toshihiko Aki, Koichi Uemura

**Affiliations:** Department of Forensic Medicine, Graduate School of Medical and Dental Sciences, Tokyo Medical and Dental University (TMDU), 1-5-45 Yushima, Bunkyo-ku, Tokyo, 113-8519 Japan

**Keywords:** Biochemistry, Cell biology, Pathogenesis

## Abstract

Abuse of the potent psychostimulant cocaine is widely established to have cardiovascular consequences. The cardiotoxicity of cocaine is mainly associated with oxidative stress and mitochondrial dysfunction. Mitochondrial dynamics and biogenesis, as well as the mitochondrial unfolded protein response (UPR^mt^), guarantee cardiac mitochondrial homeostasis. Collectively, these mechanisms act to protect against stress, injury, and the detrimental effects of chemicals on mitochondria. In this study, we examined the effects of cocaine on cardiac mitochondrial dynamics, biogenesis, and UPR^mt^ in vivo. Rats administered cocaine via the tail vein at a dose of 20 mg/kg/day for 7 days showed no structural changes in the myocardium, but electron microscopy revealed a significant increase in the number of cardiac mitochondria. Correspondingly, the expressions of the mitochondrial fission gene and mitochondrial biogenesis were increased after cocaine administration. Significant increase in the expression and nuclear translocation of activating transcription factor 5, the major active regulator of UPR^mt^, were observed after cocaine administration. Accordingly, our findings show that before any structural changes are observable in the myocardium, cocaine alters mitochondrial dynamics, elevates mitochondrial biogenesis, and induces the activation of UPR^mt^. These alterations might reflect cardiac mitochondrial compensation to protect against the cardiotoxicity of cocaine.

## Introduction

Cocaine (benzoylmethylecgonine), a strongly addictive psychostimulant, is the second most commonly used recreational drug in the world^1^. Sufficient evidence shows that the use of cocaine is related to a series of dangerous cardiovascular consequences, such as coronary artery spasm^2^, dysrhythmia^3,4^, hypertension^5^, left ventricular hypertrophy^6^, and myocardial infarction^7,8^. In addition to the anesthetic effect of cocaine by blocking sodium channels, the cocaine-induced cardiovascular disorders are mainly due to its potent sympathomimetic effect, which is achieved by the blockage of catecholamine reuptake at neuronal presynaptic terminals^9,10^. This allows cocaine not only to escalate the myocardial oxygen demand, but also, simultaneously, to decrease the myocardial oxygen flow^11,12^. In general, oxidative stress and mitochondrial dysfunction in cardiomyocytes are believed to play pivotal roles in cocaine-induced cardiotoxicity^13–17^.

Cardiac mitochondria, which occupy 20–40% of the cardiac cellular volume, are indispensable for producing the copious amounts of ATP required to sustain the unremitting contraction of the myocardium^18–20^. Moreover, mitochondria are implicated in the maintenance of intracellular Ca^2+^ homeostasis, the regulation of cell death and survival, and are the primary origin of the reactive oxygen species (ROS) that trigger oxidative stress when overproduced^21–23^. Studies have found that mitochondria play a key role in cardiovascular pathogenesis, such as dysrhythmia and heart failure^24,25^. Thence, the homeostasis and normal function of mitochondria are crucial for the ideal operation of the heart, and these are sustained by mitochondrial dynamics and biogenesis^26^.

Mitochondrial dynamics, which refers to the continuous fusion-fission circulation of mitochondria, is regulated by a series of dynamin-related GTPases^27,28^. Briefly, optic atrophy 1 (OPA1) and mitofusin (MFN) 1 and 2 facilitate mitochondrial fusion^27,29^. On the other hand, dynamin-related protein 1 (Drp1), which plays a leading role in mitochondrial fission, is phosphorylated and recruited to the fission point. Phospho-Drp1 (p-Drp1) then promotes the formation of a membrane constriction ring by reactions with receptors and Drp1 oligomerization, which then leads to the completion of mitochondrial fission^28,30,31^. Mitochondrial biogenesis, on the other hand, is driven by peroxisome proliferator-activated receptor (PPAR) γ coactivator 1α (PGC-1α)^32^. Effectors of PGC-1, such as PPARα, as well as transcription factors such as nuclear respiratory factor 1 (NRF1) and mitochondrial transcription factor A (TFAM) also participate in the regulation of mitochondrial biogenesis^33^. Mitochondrial dynamics and biogenesis form a delicate coping mechanism to maintain the optimal energic output of mitochondria in response to different environments, including increased oxidative stress and toxic conditions^27,28,34,35^. They are closely associated with the homeostasis of the cardiovascular system and an altered balance of mitochondrial dynamics can lead to pathological cardiac remodeling and cardiac pathogenesis^19,36–38^. Accordingly, the status of mitochondrial dynamics and biogenesis is fascinating when against the cardiotoxicity of cocaine. Recently, the protective role of the mitochondrial unfolded protein response (UPR^mt^) in cardiac pathophysiology has drawn increasing attention. During the accumulation of mitochondrial stress, activated UPR^mt^ promotes the recovery of the oxidative phosphorylation machinery^39^, restores proteostasis^40^, and detoxifies excess ROS^41^, accomplished by the translocation of its major regulator, activating transcription factor 5 (ATF5), and the activation of mitochondrial resident chaperones and proteases^42,43^. It remains unrevealed whether UPR^mt^ participates in resistance to the cardiotoxicity of cocaine.

Here, we investigate whether cocaine affects cardiac mitochondrial dynamics and biogenesis in vivo. After rats were administered 20 mg/kg/day cocaine via the tail vein for 7 days, we found strengthened cardiac mitochondrial fission and biogenesis, as well as the recruitment of PPARα, while the expressions of mitochondrial fusion factors were decreased. In addition, UPR^mt^ was shown to be activated, although only a panel of its downstream effector gene expressions were indeed upregulated. We believe that this is the first in vivo study showing the altered mitochondrial dynamics and biogenesis, as well as the involvement of PPARα and UPR^mt^, in cardiomyocytes after cocaine administration.

## Materials and methods

All methods were carried out in accordance with relevant guidelines and regulations.

### Animals and cocaine administration

All animal experiments were approved by the Institutional Animal Care and Use Committee of Tokyo Medical and Dental University. All animal work was performed in accordance with Animal Research: Reporting of In Vivo Experiments (ARRIVE) guidelines and regulations. Male Sprague Dawley rats (8 weeks old) were randomly divided into 3 groups (n = 4, respectively) and administered saline (control group) or cocaine [low (5 mg/kg)/ high (20 mg/kg) dose cocaine group] in the first-round experiments. In second-round experiments to further examine expressions of oxidative phosphorylation system proteins, complex I activity, the protein expressions of UPR^mt^ factors and PPARα, and to obtain samples for transmission electron microscopy, a control group (n = 4) and a high dose cocaine group (n = 7) were established under the same condition as the first-round experiments. Findings of the two rounds of experiments are shown collectively to provide a comprehensive demonstration of the consequences of cocaine cardiotoxicity.

Dose conversions from humans to rats were made to establish the conditions of 5 and 20 mg/kg/day to represent typical serum concentrations for recreational cocaine abusers and beneath the concentrations for acute cocaine poisoning overdose^44,45^. Cocaine hydrochloride (Shionogi & Co., Ltd., Osaka, Japan) was dissolved in saline and administered intravenously via the tail vein (5 mg/kg/day for the low dose cocaine group, 20 mg/kg/day for the high dose cocaine group) for 7 days. Control rats received the same volume of saline injected via the tail vein on the same schedule. The rats were sacrificed by an overdose of sodium pentobarbital (40 mg/kg, intraperitoneally) 24 h after the last administration. The hearts were immediately harvested and placed in phosphate-buffered saline solution (PBS, 4 °C, pH 7.4). Subsequently, the left ventricles were evenly divided for further histological and biochemical analyses.

### Histological analysis and immunohistochemical staining

The left ventricular specimens for microscopy were fixed in 4% paraformaldehyde, embedded in paraffin, and 2.5 µm-thick sections were prepared. Hematoxylin–eosin (H&E) and Elastica-Masson-Goldner (EMG) staining were performed for histological analysis. Immunohistochemical staining of PPARα and ATF5 were conducted. Briefly, sections were deparaffinized in xylene, rehydrated with a series of graded ethanol, and heated in 10 mM citrate buffer (pH 6.0) for antigen retrieval. 3% hydrogen peroxide was applied afterward to quench the activity of endogenous peroxidase. The sections were then blocked with 0.5% bovine serum albumin serum in PBS. Subsequently, the sections were incubated with rabbit anti-ATF5 monoclonal antibody (diluted 100-fold; ab184923, Abcam, Cambridge, UK) or mouse anti-PPARα monoclonal antibody (diluted 100-fold; sc-398394, Santa Cruz Biotechnology, Inc., USA) overnight at 4 °C, followed by visualization of antigens by use of Histofine simple stain MAX-PO (Multi) (Nichirei Biosciences Inc., Tokyo, Japan) using diaminobenzidine (Nichirei Biosciences Inc.) as a substrate. Sections incubated in PBS in place of the primary antibodies were used as controls for immunostaining procedures.

### Mitochondrial electron transport chain activity detection

The left ventricle specimens for mitochondrial complex I enzyme activity analysis were immediately frozen to − 80 °C. Mitochondria were extracted from the specimens using a Mitochondria Isolation Kit (ab 110168, Abcam). The protein concentrations of the extracts were determined by Bicinchoninic Acid protein assay and adjusted to 100 µg/mL with PBS. Subsequently, a Complex I Enzyme Activity Assay Kit (ab109721, Abcam) was used to determine the activity of the electron transport chain (ETC) following the manufacturer’s protocol. The absorbance at 450 nm was measured using a microplate reader (GloMax Discover System GM3000, Promega Corporation, USA).

### Immunoblotting analysis

The left ventricle specimens for immunoblotting were lysed in STE buffer (320 mM sucrose, 10 mM Tris–HCl, 5 mM EDTA, 50 mM NaF, 2 mM Na_3_VO_4_, and protease inhibitor cocktail (Roche Diagnostics, Mannheim, Germany)), and the lysates were subjected to SDS–polyacrylamide gel electrophoresis. Immunoblotting was then performed with voltage-dependent anion channel 2 (VDAC2) antibody (#9412, Cell Signaling Technology, Beverly, MA, USA), the translocase of the outer mitochondrial membrane 20 (TOM20) antibody, (#42406, Cell Signaling Technology), total OXPHOS Rodent WB antibody cocktail (ab110413, Abcam), anti-PGC-1 polyclonal antibody (#AB3242, EMD Millipore Corporation, CA, USA), anti-NRF1 antibody (sc-33771, Santa Cruz Biotechnology, Inc.), anti- TFAM antibody (sc-23588, Santa Cruz Biotechnology, Inc.), anti-PPARα monoclonal antibody (Santa Cruz Biotechnology, Inc.), anti-ATF5 rabbit monoclonal antibody (Abcam), anti-CHOP mouse monoclonal antibody (#2895, Cell Signaling Technology), and anti-actin antibody (A2066, Sigma-Aldrich, St. Louis, MO, USA). Antigens were visualized by peroxidase-bonded anti-Mouse or rabbit IgG secondary antibodies (Promega Corporation, USA) and enhanced chemiluminescence reagents (Thermo Fisher Scientific, USA). The band densities were quantified by CS analyzer 4 image analyzing software (Atto, Tokyo, Japan).

### Transmission electron microscopy

The left ventricle specimens for electron microscopy were washed with 0.1 M phosphate buffer (PB) and fixed with 4.5% paraformaldehyde and 2.5% glutaraldehyde in PB. After incubated in 1% osmium tetroxide for 2 h, the fixed specimens were dehydrated in a series of graded ethanol and embedded in Epon epoxy resin. Ultrathin sections of the embedded specimens were stained with uranyl acetate and lead citrate and observed under a transmission electron microscope (H7100, Hitachi, Japan) and AMT Advantage-HS CCD camera (AMT, Woburn, USA). The number of mitochondria per field (magnitude: 10,000×) was counted from 10 electron micrographs in each group. Randomly selected cross-sectional areas of mitochondria (n = 100 for each group) in cross-sectional views of cardiac myofibrils were counted using ImageJ software (ver1.53).

### Real-time reverse transcriptase-mediated PCR analysis

Real-time reverse transcriptase-mediated PCR analysis was conducted as described previously^46^. The primers used are listed in Supplementary Table S1.

### Statistical analysis

Student’s t-test and Dunnett’s test were used to assess statistical significance throughout this study. Statistical differences were considered significant at *p* < 0.05.

## Results

### No structural changes in the myocardium after 7 days cocaine administration via the tail vein

**Figure 1:**
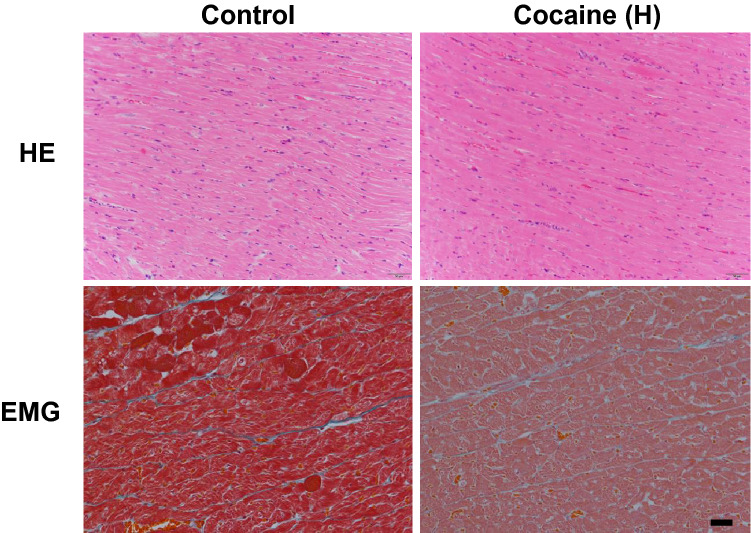
Microscopy of myocardium after cocaine administration. Hematoxylin and Eosin (HE) and Elastica-Masson–Goldner (EMG) staining of rat left ventricular samples from control and cocaine high dose groups, cocaine (H), cocaine high dose group, scale bar = 50 µm.

Cocaine has been reported to induce cardiovascular disorders in animal models via intraperitoneal administration. To assess the effect of cocaine on the myocardium in our model using tail vein administration, H&E and EMG staining of left ventricular specimens from the control group and cocaine group rats were conducted (Fig. 1). The myocardium of the cocaine group rats showed no signs of hypertrophy since the size and arrangement of myocardial fibers were retained after 20 mg/kg/day administration for 7 days. There was also no observable inflammatory infiltration or fibrosis deposition in the myocardium in the cocaine group. No significant pathological changes other than congestion were observed in the microphotographs of the cocaine high dose group. As a result, the histological findings suggest a basically normal myocardium after cocaine administration via the tail vein.

### Unimpaired cardiac mitochondrial activity and elevated mitochondrial fission after 7 days cocaine administration via tail vein

**Figure 2:**
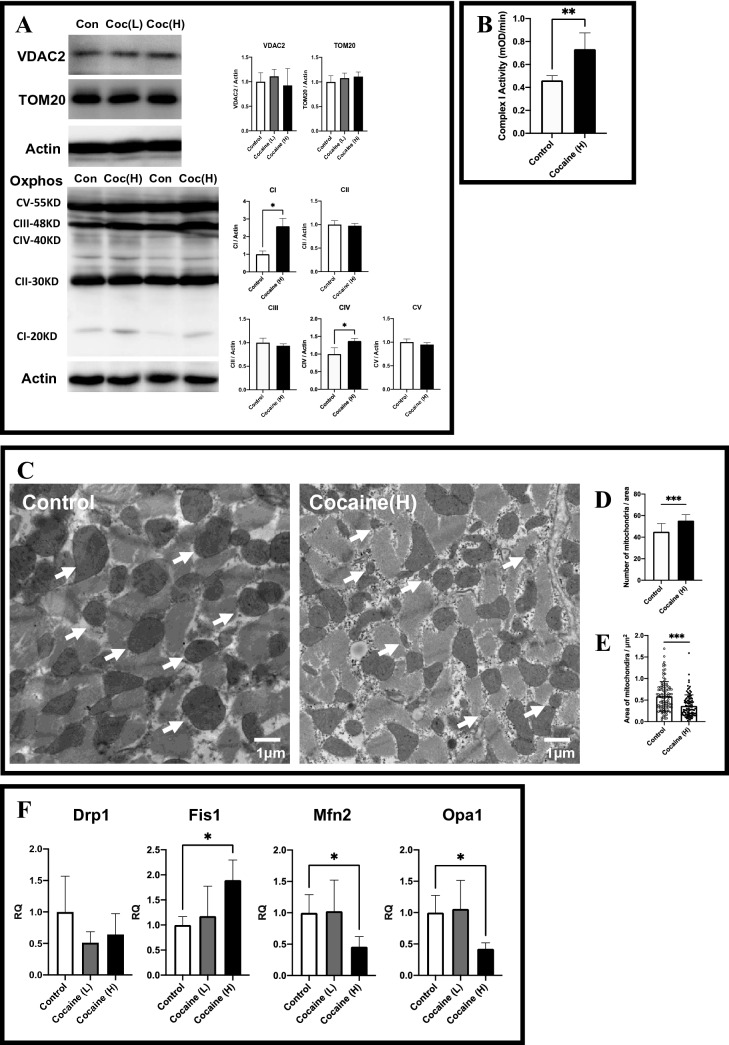
Maintained cardiac mitochondrial activity and elevated mitochondrial fission after cocaine administration. (**A**) Protein expressions of voltage-dependent anion-selective channel protein 2 (VDAC2), translocase of outer mitochondrial membrane 20 (TOM20), and mitochondria oxidative phosphorylation subunits (Oxphos) in rat left ventricle after cocaine administration. Levels of actin were served as an internal control. (**B**) Detection of endogenous Cox I activity in cardiac mitochondria isolated from myocardium samples from control and cocaine high dose group rats. (**C**) Transmission electron micrographs of left ventricular samples from control and cocaine group rats. Magnification, × 10,000. White arrows indicate mitochondria. (**D**) The number of mitochondria per field in control and cocaine group rats. Each bar represents the mean and S.D. of 10 areas. (**E**) The area of mitochondria in control and cocaine group rats. Comparison of the distribution of mitochondrial area in control and cocaine group rats is shown. Over 100 mitochondria were measured in each group and the area was measured in cross-sectional views of cardiac myofibrils. (**F**) Dynamin-related protein 1 (Drp1), mitochondrial fission 1 protein (Fis1), Mitofusin 2 (Mfn2), and Optic atrophy type 1 (Opa1) expressions after cocaine administration as examined by qPCR. GAPDH levels served as an endogenous control. Each bar represents mean and S.D. *p < 0.05, **p < 0.01, ***p < 0.001, *C I–V* complex I–V. *Con* control group, *Coc (L)* cocaine low dose group, *Coc (H)* cocaine high dose group, *RQ* relative quantification.

Next, we determined the effect of cocaine on cardiac mitochondrial status and function. Immunoblotting of mitochondria oxidative phosphorylation subunit proteins showed an increase in the complex I protein. Apart from complex I and IV proteins, the expressions of complex II, complex III, complex V, VDAC2, and TOM20 showed no significant changes after cocaine administration (Fig. 2A). To obtain a better understanding of cardiac mitochondrial function after cocaine administration, endogenous complex I activity assays using left ventricle samples from the control and cocaine high dose groups were conducted. Consistent with the immunoblotting result, the complex I activity of the cocaine high dose group myocardium was significantly elevated compared to the activity of the control group (Fig. 2B).

Owing to the seemingly normal mitochondrial status and function, transmission electron microscopy was conducted to acquire a direct observation of cardiac mitochondrial morphological changes after cocaine administration. As shown in Fig. 2C, the number of cardiac mitochondria was notably increased in the cocaine high dose group as compared to the control group (Fig. 2D). At the same time, the cross-sectional area of the cardiac mitochondria as checked on cross-sections of the myocardium decreased remarkably after cocaine administration (Fig. 2E).

Given the indications that the mitochondrial activity as well as homeostasis of the cardiomyocytes was maintained with the increased number of mitochondria, we suspect the cardiac mitochondrial dynamics were affected by cocaine administration. To examine this possibility, the relative levels of transcripts that participate in mitochondrial dynamics were evaluated by qPCR. Expression of the mitochondrial fission-related gene *Fis1* showed an apparent increase, while the expressions of mitochondrial fusion-related genes *Mfn2* and *Opa1* showed significant decreases in response to cocaine administration (Fig. 2F). These findings indicate that activated mitochondrial fission might be involved in the maintenance of mitochondrial activity as well as cardiac homeostasis.

### Activation of mitochondrial biogenesis and nuclear translocation of PPARα after 7 days cocaine administration via tail vein

**Figure 3:**
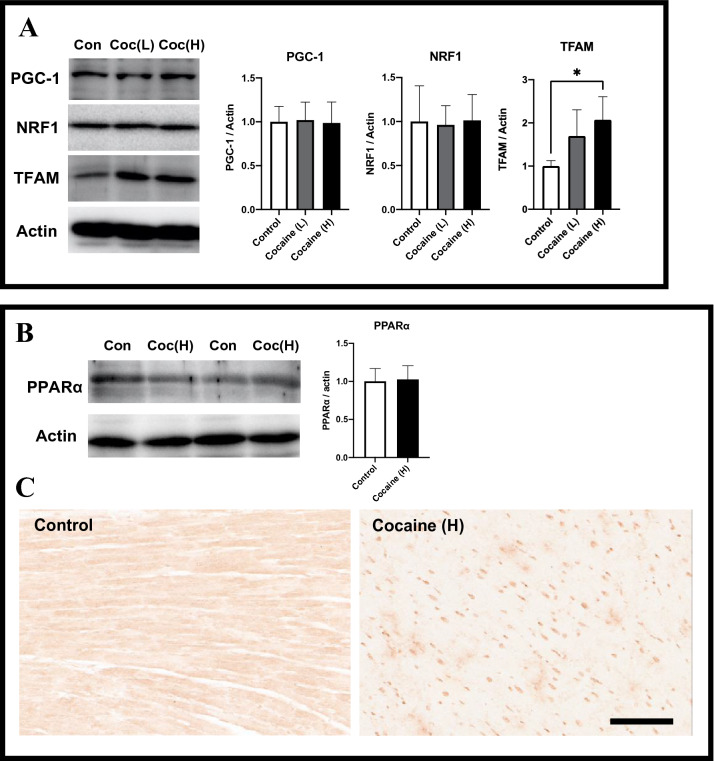
Activation of TFAM and nuclear translocation of PPARα after cocaine administration. (**A**) Expressions of peroxisome proliferator-activated receptor-gamma coactivator 1 (PGC1), nuclear respiratory factor 1 (NRF1), and mitochondrial transcription factor A (TFAM) in rat left ventricle after cocaine administration. (**B**) Expression of peroxisome proliferator-activated receptor α (PPARα) in rat left ventricle after cocaine administration. Actin levels served as an internal control. Each bar represents mean and S.D. *p < 0.05. (**C**) Immunohistochemistry of PPARα in rat left ventricular samples from control and cocaine high dose groups showed apparent nuclear translocation of positive staining after cocaine administration. Scale bar = 100 µm.

Mitochondrial biogenesis was another suspected participator in the maintenance of mitochondrial activity. We therefore examined the expressions of proteins related to cardiac mitochondrial biogenesis by immunoblotting. The expression of TFAM in the cocaine high dose group, was significantly increased as compared to its expression in the control group, whereas the expressions of PGC-1 and NRF1 showed no significant changes after cocaine administration (Fig. 3A). These findings indicate activated biogenesis and the likely activation of the NRF1-TFAM axis. Consequently, we examined PPARα, an effector of PGC-1, by immunoblotting and immunohistochemistry. Although immunoblot analysis showed no significant change in PPARα expression in the high dose cocaine group as compared to the control group (Fig. 3B), an apparent nuclear translocation of PPARα after cocaine administration was observed by immunohistochemistry (Fig. 3C). These observations suggest the activation of mitochondrial biogenesis and the recruitment of PPARα after cocaine administration.

### Activation of cardiac mitochondrial unfolded protein response after 7 days cocaine administration via tail vein

**Figure 4:**
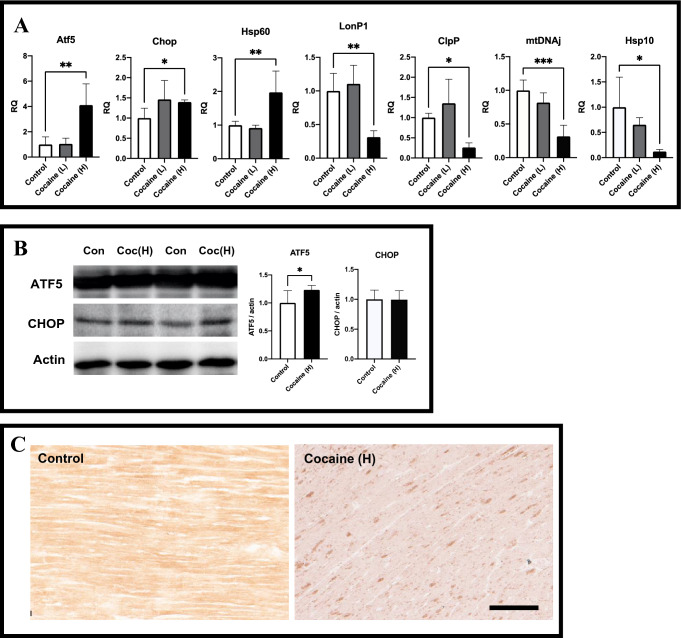
Partial activation of cardiac mitochondrial unfolded protein response after cocaine administration. (**A**) The expressions of activating transcription factor 5 (Atf5), CCAT-enhancer-binding protein homologous protein (CHOP), heat shock 60 kDa protein 1 (Hsp60), Lon protease homolog, mitochondrial (LonP1), ATP-dependent Clp protease proteolytic subunit (ClpP), mitochondrial pre-sequence translocase-associated motor complex protein (mtDNAj), and heat shock 10 kDa protein (Hsp10) after cocaine administration as examined by qPCR. GAPDH levels served as an endogenous control. RQ, relative quantification. (**B**) Expressions of ATF5 and CHOP in rat left ventricle after cocaine administration. Actin levels served as an internal control. Each bar represents mean and S.D. Coc (H), cocaine high dose group, *p < 0.05, **p < 0.01, ***p < 0.001. (**C**) Immunohistochemistry of ATF5 in rat left ventricular samples from control and cocaine high dose groups showed an apparent nuclear translocation of positive staining after cocaine administration. cocaine (H), cocaine high dose group. Scale bar = 100 µm.

Finally, to clarify the role of UPR^mt^ in our observed effects of cocaine on cardiac mitochondria, UPR^mt^-related regulators and factors were examined by qPCR, immunoblotting, and immunohistochemistry. The expressions of the major regulator of UPR^mt^ activation gene *ATF5*, transcription factor gene *CHOP*, and mitochondrial chaperone gene *HSP60* were all remarkably increased; however, the genes for other factors such as *LONP1*, *CLPP*, *mtDnaJ*, and *HSP10* were rather decreased (Fig. 4A). Further immunoblotting of ATF5 and CHOP revealed the same increase in ATF5 expression (Fig. 4B). The immunohistochemistry of ATF5 further revealed the nuclear translocation of ATF5 in cardiomyocytes after cocaine administration (Fig. 4C), consistent with the report that ATF5, once activated, undergoes nuclear translocation to signal UPR^mt^^42^. These findings indicate the occurrence of cardiac UPR^mt^ after 7 days cocaine administration via tail vein, although it seems that UPR^mt^ occured in an incomplete or atypical manner as compared with typical UPR^mt^.

## Discussion

Abundant evidence implicates cocaine abuse in cardiovascular disease. The clinical short-term consequences of cocaine intoxication include acute myocardial infarction^10^, arterial wall inflammation^47^, endocarditis^48^, interstitial inflammatory infiltration, and fibrosis^49^. Animal cocaine models in which cocaine is delivered by intraperitoneal injection also reproduce cardiomyopathies such as inflammatory infiltration^50^, contraction band necrosis^51^, and left ventricular dysfunction^13^. Intriguingly, no observation of notable structural changes was found in rat myocardium after 7 days of cocaine administration (20 mg/kg/day) via tail vein in our experiments. This might be owing to the relatively short experimental period of our study. Additionally, although there are no comparative studies on toxicity of cocaine administered via different routes, one report has suggested a lower toxicity of gold nanoparticles administered via the tail vein as compared to intraperitoneal injection^52^. As a result, it is also possible that our negative histological findings of structural changes in myocardium were due to the influence of the different administration route of cocaine as compared with previous researches. We further investigated the status of cardiac mitochondria after cocaine administration because mitochondrial dysfunction is one of the hallmarks of cocaine cardiotoxicity. These examinations revealed normally reserved mitochondrial status and function. The function of complex I, the first and largest enzyme of ETC, and most of its core subunits, which are mitochondrially encoded proteins^53^, were elevated rather than impaired after 7 days of cocaine administration via the tail vein. It has been repeatedly reported that cocaine-induced oxidative stress increases the production of ROS, damages the ETC, suppresses the generation of ATP^54^, as well as compromises the antioxidative system^50,54^, and all of these eventually results in cardiac mitochondrial dysfunction^17,55^. Animal models of cocaine abuse have also revealed an extensively decreased expression of mitochondrial genes and proteins^56,57^. The rare but meaningful findings in our study showed a still normally maintained mitochondrial status after cocaine administration, which suggests that a mitochondria-targeted self-compensation mechanism against cocaine cardiotoxicity was activated prior to the occurrence of any structural changes in myocardium.

As stated above, mechanisms such as mitochondrial dynamics, biogenesis, and UPR^mt^ help to preserve mitochondrial homeostasis in cardiomyocytes and act protectively against detrimental effects. In particular, mitochondrial dynamics and biogenesis take part in determining mitochondrial quality and abundance, thereby maintaining adequate myocardial function^26,27,58–61^. Elevated mitochondrial fission and biogenesis meet the increased energy demand of cardiac myocytes under stress or ischemic injury or the harmful effects of chemicals^26,58,62^. We discovered that it is the same circumstance in cardiac mitochondria after cocaine administration. In this study, we found the activations of mitochondrial fission and biogenesis, together with downregulated mitochondrial fusion gene expression. These effects may act together to profoundly increase the number of mitochondria, as we observed by electron microscopy, so as to maintain normal cardiac function under the detrimental effects of cocaine. Mitochondrial Fis1 recruitment has been demonstrated as an early event in the cardiac pathological process^63,64^, and the elevated fission machinery ultimately results in cardiomyopathy^20,26^. It is consistent with our view of the mitochondrial compensatory stage ahead of any pathological changes. In addition, during pathological cardiac remodeling, TFAM protects the mitochondrial DNA from mutation, and its overexpression is beneficial to the recovery of cardiac function^65^. It is likely that this protective effect of TFAM might also participate in the specific upregulation of mitochondrial DNA and preserved function of complex I seen in this study. Meanwhile, as the effector of PGC-1, PPARα also participates in mitochondrial biogenesis and homeostasis because of its central role in fatty acid oxidation and other lipid metabolism events^66–68^. However, the involvement of PPARα in the cardiotoxicity of cocaine has not been illustrated. Our immunohistochemical observation indicates the recruitment of PPARα in the myocardium after cocaine administration. This might be related to our observation of elevated mitochondrial biogenesis in cocaine group rats and implies a potential role of PPARα in resistance to the cardiotoxicity of cocaine. UPR^mt^ is another potential protective mechanism to explain the unimpaired mitochondrial function we observed. UPR^mt^ activation has been shown to be important for the normal function of cardiac myocytes^43^. Among numerous involved factors, ATF5 should be involved in the UPR^mt^ cardioprotective effects against stress, cardiac dysfunction, and pathological remodeling, because its activation and nuclear translocation trigger the downstream responses of UPR^mt^^69,70^. CHOP and Hsp60, on the other hand, play key roles in maintaining mitochondrial homeostasis in UPR^mt^^71^. Our examination of UPR^mt^ revealed an interesting phenomenon that instead of all UPR^mt^ factors being collectively upregulated after cocaine administration, the expressions of several UPR^mt^ factors decreased significantly. It has been reported that Lon regulates mitochondrial transcription by selectively degrading TFAM^72^, and the absence of *CLPP* reduces mitochondrial cardiomyopathy^73^. These findings suggest that the downregulation of *LONP1* and *CLPP* that we observed might also play a protective role against cocaine cardiotoxicity. Nevertheless, further research will be required in order to fully understand the paradoxical expression of UPR^mt^ factors that we observed. So far, our findings indicate that UPR^mt^ participates in cardiac mitochondrial protection against the cardiotoxicity of cocaine, even if not complete and collectively activated at an early stage.

In conclusion, we demonstrated that 7-day 20 mg/kg/day cocaine administration to rats via the tail vein results in elevated cardiac mitochondrial fission and biogenesis, the recruitment of PPARα, and the activation of mitochondrial protecting UPR^mt^ in the myocardium. The limit of this study is the insufficient data of cardiac and mitochondrial functional changes, as well as stress accumulation after cocaine administration, which necessitates more functional assessments of heart and mitochondria in following studies, such as echocardiography, respiration profiles, and ATP production rates. It is worth noticing that the differences might present among individual models, but our findings should be beneficial for an advanced understanding of the cardiotoxicity of cocaine, especially the early events following cocaine exposure.

## Supplementary Information

Supplementary Information.
