# Supplementary material for: Altered cardiac mitochondrial dynamics and biogenesis in rat after short-term cocaine administration
Source: Sci Rep. 2021 Dec 16;11:24129. doi: 10.1038/s41598-021-03631-y (PMC8677764; doi:10.1038/s41598-021-03631-y)
Supplement: Supplementary file 1 — Supplementary Information. [file 41598_2021_3631_MOESM1_ESM.pptx]

## Slide 1
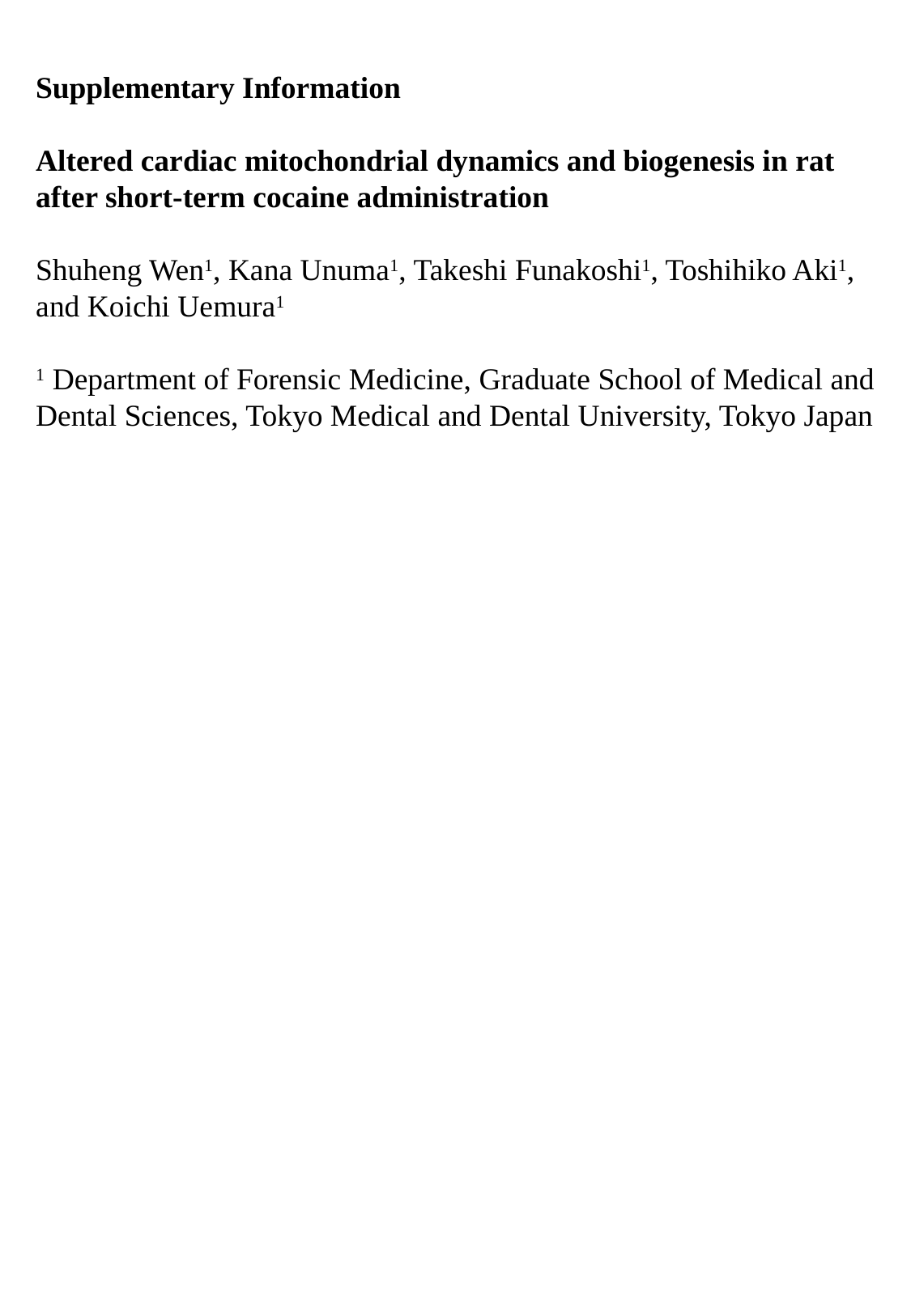

Supplementary Information
Altered cardiac mitochondrial dynamics and biogenesis in rat after short-term cocaine administration
Shuheng Wen1, Kana Unuma1, Takeshi Funakoshi1, Toshihiko Aki1, and Koichi Uemura1
1 Department of Forensic Medicine, Graduate School of Medical and Dental Sciences, Tokyo Medical and Dental University, Tokyo Japan

## Slide 2
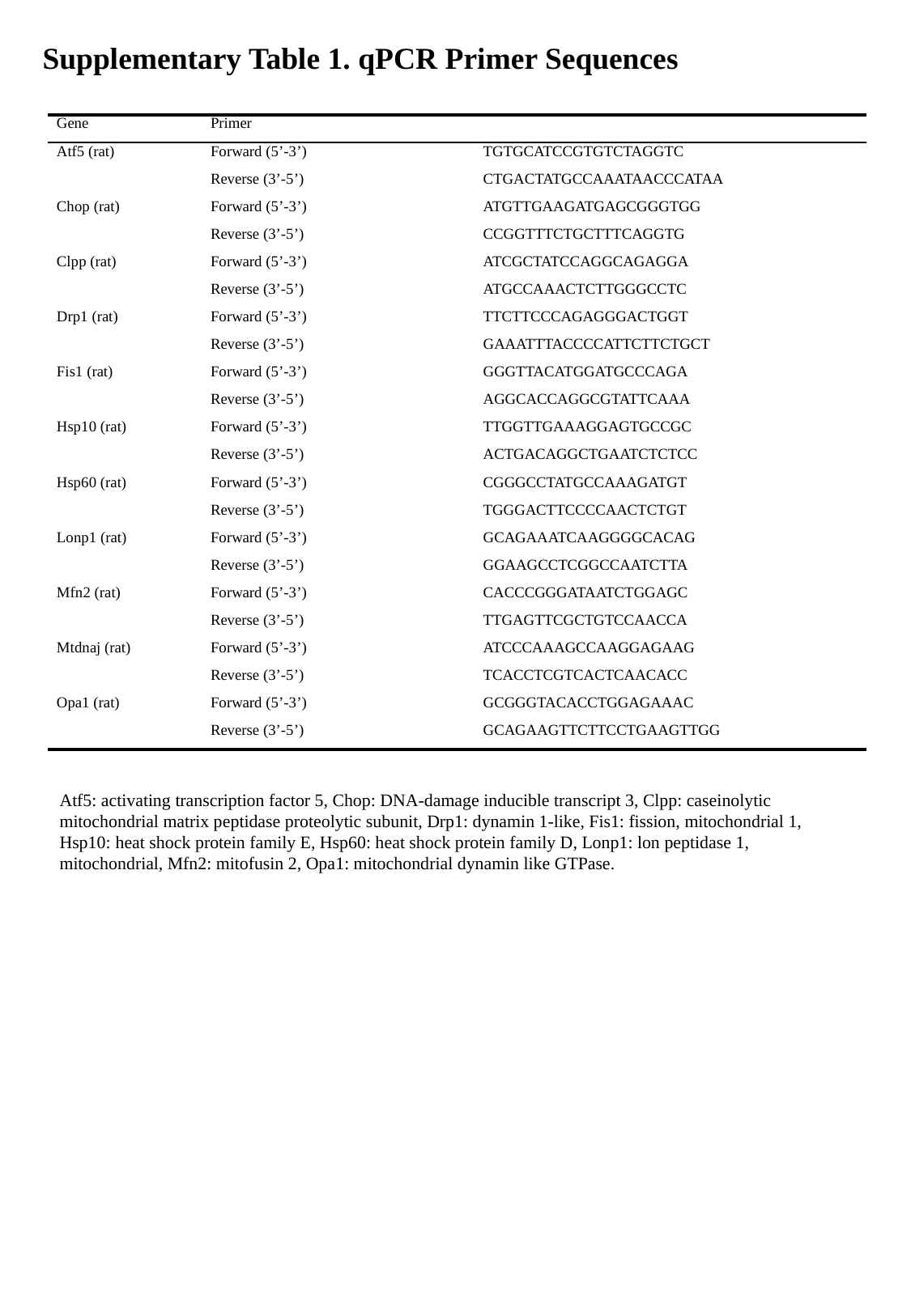

Supplementary Table 1. qPCR Primer Sequences
| Gene | Primer | |
| --- | --- | --- |
| Atf5 (rat) | Forward (5’-3’) | TGTGCATCCGTGTCTAGGTC |
| | Reverse (3’-5’) | CTGACTATGCCAAATAACCCATAA |
| Chop (rat) | Forward (5’-3’) | ATGTTGAAGATGAGCGGGTGG |
| | Reverse (3’-5’) | CCGGTTTCTGCTTTCAGGTG |
| Clpp (rat) | Forward (5’-3’) | ATCGCTATCCAGGCAGAGGA |
| | Reverse (3’-5’) | ATGCCAAACTCTTGGGCCTC |
| Drp1 (rat) | Forward (5’-3’) | TTCTTCCCAGAGGGACTGGT |
| | Reverse (3’-5’) | GAAATTTACCCCATTCTTCTGCT |
| Fis1 (rat) | Forward (5’-3’) | GGGTTACATGGATGCCCAGA |
| | Reverse (3’-5’) | AGGCACCAGGCGTATTCAAA |
| Hsp10 (rat) | Forward (5’-3’) | TTGGTTGAAAGGAGTGCCGC |
| | Reverse (3’-5’) | ACTGACAGGCTGAATCTCTCC |
| Hsp60 (rat) | Forward (5’-3’) | CGGGCCTATGCCAAAGATGT |
| | Reverse (3’-5’) | TGGGACTTCCCCAACTCTGT |
| Lonp1 (rat) | Forward (5’-3’) | GCAGAAATCAAGGGGCACAG |
| | Reverse (3’-5’) | GGAAGCCTCGGCCAATCTTA |
| Mfn2 (rat) | Forward (5’-3’) | CACCCGGGATAATCTGGAGC |
| | Reverse (3’-5’) | TTGAGTTCGCTGTCCAACCA |
| Mtdnaj (rat) | Forward (5’-3’) | ATCCCAAAGCCAAGGAGAAG |
| | Reverse (3’-5’) | TCACCTCGTCACTCAACACC |
| Opa1 (rat) | Forward (5’-3’) | GCGGGTACACCTGGAGAAAC |
| | Reverse (3’-5’) | GCAGAAGTTCTTCCTGAAGTTGG |
Atf5: activating transcription factor 5, Chop: DNA-damage inducible transcript 3, Clpp: caseinolytic mitochondrial matrix peptidase proteolytic subunit, Drp1: dynamin 1-like, Fis1: fission, mitochondrial 1, Hsp10: heat shock protein family E, Hsp60: heat shock protein family D, Lonp1: lon peptidase 1, mitochondrial, Mfn2: mitofusin 2, Opa1: mitochondrial dynamin like GTPase.

## Slide 3
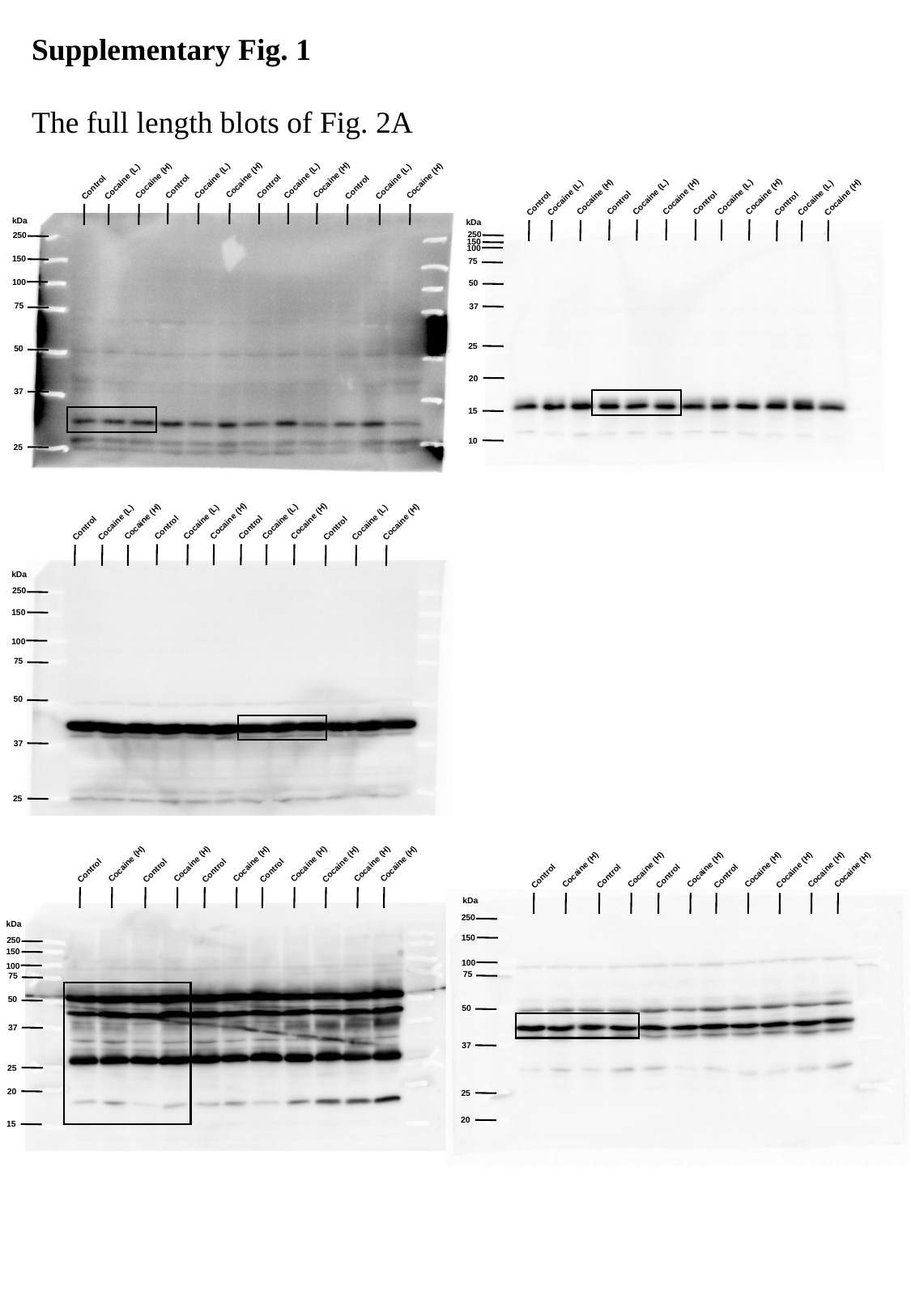

Supplementary Fig. 1
The full length blots of Fig. 2A
Cocaine (H)
Cocaine (H)
Cocaine (H)
Cocaine (H)
Cocaine (L)
Cocaine (L)
Cocaine (L)
Cocaine (L)
Control
Control
Control
Control
Cocaine (H)
Cocaine (H)
Cocaine (H)
Cocaine (H)
Cocaine (L)
Cocaine (L)
Cocaine (L)
Cocaine (L)
Control
Control
Control
Control
kDa
kDa
250
250
150
100
150
75
100
50
75
37
25
50
20
37
15
10
25
Cocaine (H)
Cocaine (H)
Cocaine (H)
Cocaine (H)
Cocaine (L)
Cocaine (L)
Cocaine (L)
Cocaine (L)
Control
Control
Control
Control
kDa
250
150
100
75
50
37
25
Cocaine (H)
Cocaine (H)
Cocaine (H)
Cocaine (H)
Cocaine (H)
Cocaine (H)
Cocaine (H)
Cocaine (H)
Cocaine (H)
Cocaine (H)
Cocaine (H)
Cocaine (H)
Cocaine (H)
Cocaine (H)
Control
Control
Control
Control
Control
Control
Control
Control
kDa
250
kDa
150
250
150
100
100
75
75
50
50
37
37
25
20
25
20
15

## Slide 4
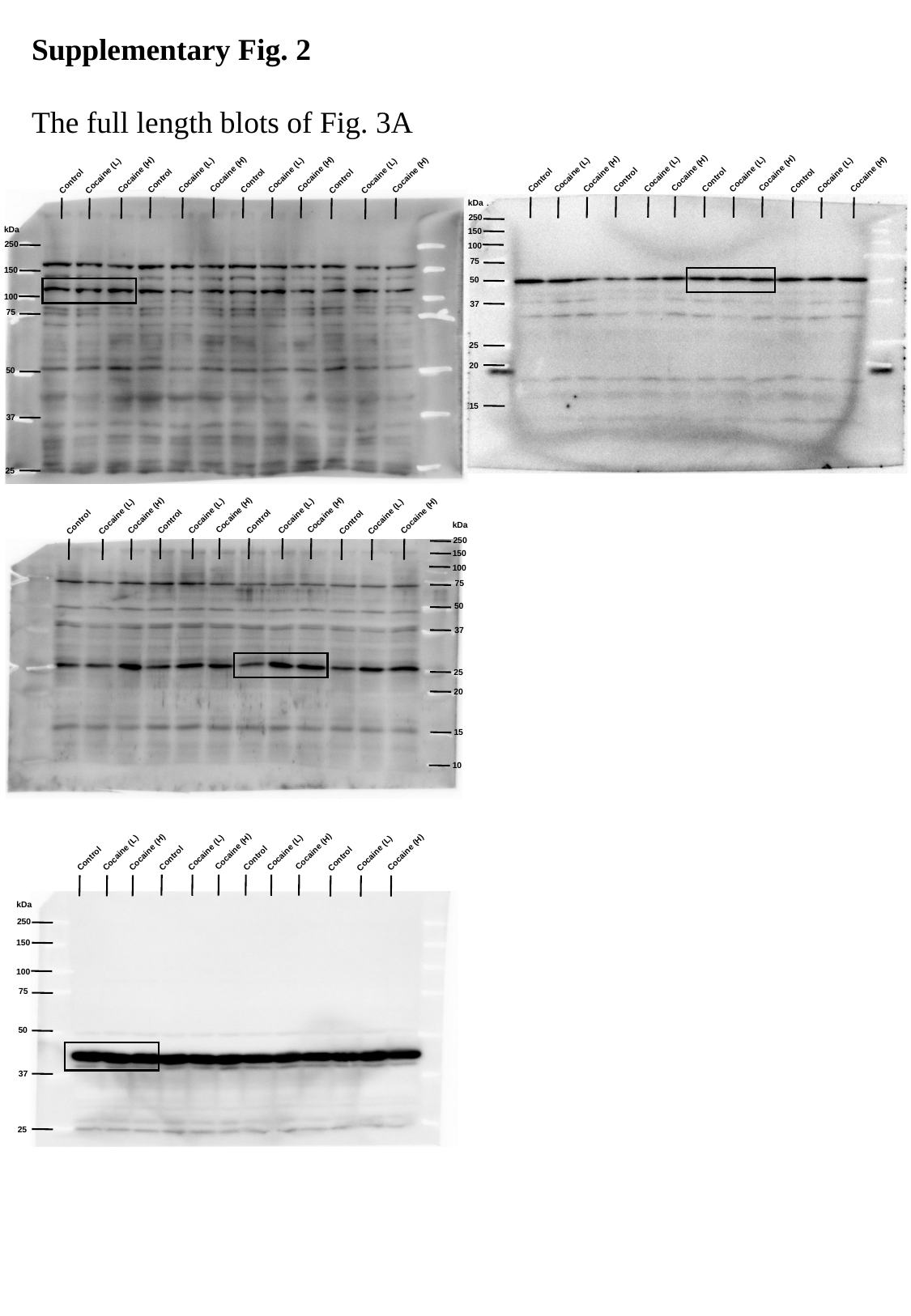

Supplementary Fig. 2
The full length blots of Fig. 3A
Cocaine (H)
Cocaine (H)
Cocaine (H)
Cocaine (H)
Cocaine (H)
Cocaine (H)
Cocaine (L)
Cocaine (L)
Cocaine (H)
Cocaine (L)
Cocaine (H)
Cocaine (L)
Cocaine (L)
Cocaine (L)
Cocaine (L)
Cocaine (L)
Control
Control
Control
Control
Control
Control
Control
Control
kDa
250
kDa
150
250
100
75
150
50
100
37
75
25
20
50
15
37
25
Cocaine (H)
Cocaine (H)
Cocaine (H)
Cocaine (H)
Cocaine (L)
Cocaine (L)
Cocaine (L)
Cocaine (L)
Control
Control
Control
Control
kDa
250
150
100
75
50
37
25
20
15
10
Cocaine (H)
Cocaine (H)
Cocaine (H)
Cocaine (H)
Cocaine (L)
Cocaine (L)
Cocaine (L)
Cocaine (L)
Control
Control
Control
Control
kDa
250
150
100
75
50
37
25

## Slide 5
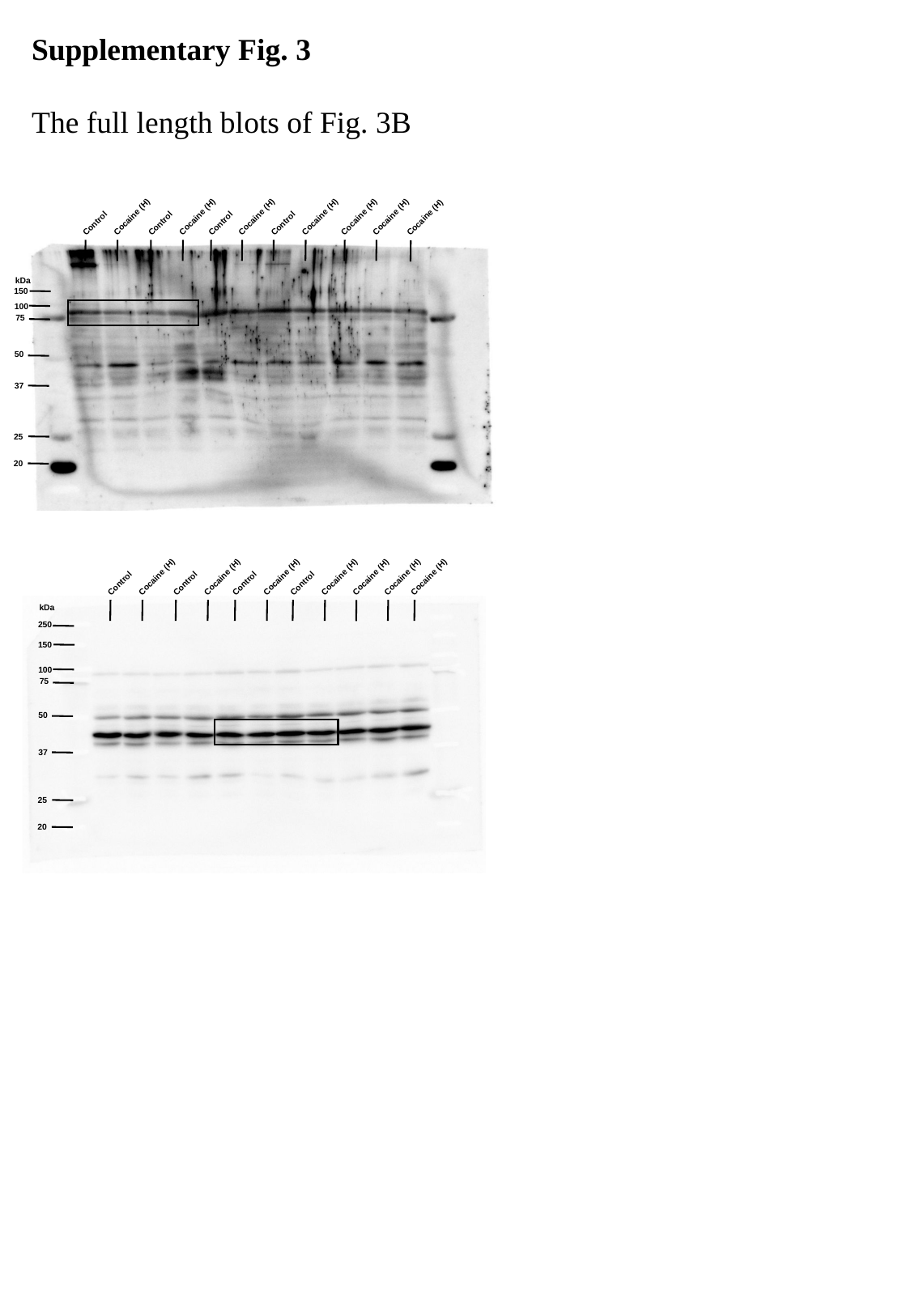

Supplementary Fig. 3
The full length blots of Fig. 3B
Cocaine (H)
Cocaine (H)
Cocaine (H)
Cocaine (H)
Cocaine (H)
Cocaine (H)
Cocaine (H)
Control
Control
Control
Control
kDa
150
100
75
50
37
25
20
Cocaine (H)
Cocaine (H)
Cocaine (H)
Cocaine (H)
Cocaine (H)
Cocaine (H)
Cocaine (H)
Control
Control
Control
Control
kDa
250
150
100
75
50
37
25
20

## Slide 6
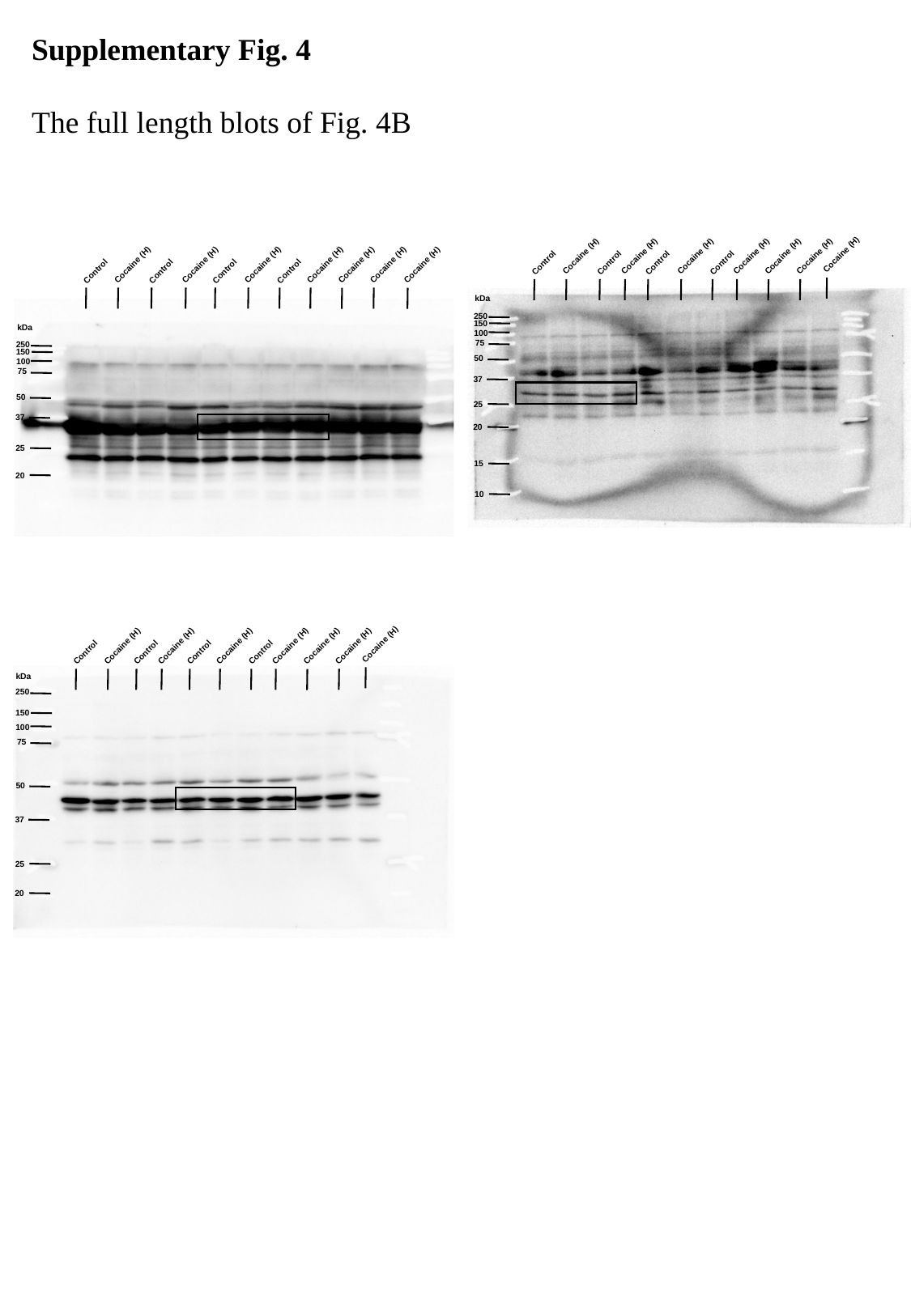

Supplementary Fig. 4
The full length blots of Fig. 4B
Cocaine (H)
Cocaine (H)
Cocaine (H)
Cocaine (H)
Cocaine (H)
Cocaine (H)
Cocaine (H)
Control
Control
Control
Control
Cocaine (H)
Cocaine (H)
Cocaine (H)
Cocaine (H)
Cocaine (H)
Cocaine (H)
Cocaine (H)
Control
Control
Control
Control
kDa
250
150
kDa
100
75
250
150
50
100
75
37
50
25
37
20
25
15
20
10
Cocaine (H)
Cocaine (H)
Cocaine (H)
Cocaine (H)
Cocaine (H)
Cocaine (H)
Cocaine (H)
Control
Control
Control
Control
kDa
250
150
100
75
50
37
25
20
